# The Treatment of Warts

**Published:** 1905-10-21

**Authors:** 


### THE TREATMENT OF WARTS.

According to Dr. J. Burdon Cooper[1], lime water acts almost as a specific in the treatment of warts and allied conditions. He first noted this in the case of a small but obstinate wart on his own thumb, which, after resisting other methods, disappeared when lime-water was taken for the relief of another ailment. Subsequently he ordered the same remedy whenever opportunity offered, all forms of local treatment being at the same time avoided, and he now reports unqualified success. The lime-water is given with a little milk after the mid-day meal, the dose being a wineglassful. The time taken for the total disappearance of the warts varies from four days to six weeks.

Another internal medicine that has been recommended in the treatment of warts is magnesium sulphate. It should be ordered in doses sufficient to produce two or three actions of the bowel every day. In a case of multiple warts distributed over the hands, forearms, and face, Dr. Vavapy[2] reported the treatment by magnesium sulphate as completely successful.

Among local applications that have been advised Dr. Morison [3] has found the following very useful: Perchloride of mercury, grains 5, and a dram of salicylic acid dissolved in an ounce of collodion. If necesary the mercurial salt may be increased up to 30 grains. The application should be applied once a day, the crust formed by the previous layer being removed before a fresh one is made.

[1] Brit. Med. Jour., August 26, 1905. [2] Clin. Jour., Vol. II. [3] Ibid.

